# Connexins in metabolic control: linking brain cellular communication to systemic homeostasis

**DOI:** 10.1093/lifemedi/lnag018

**Published:** 2026-05-11

**Authors:** Yiyi Zhu, Hui Chen, Dan Li, Alexei Verkhratsky, Chenju Yi

**Affiliations:** Department of Geriatrics, The Seventh Affiliated Hospital of Sun Yat-sen University, Shenzhen 518107, China; School of Life Sciences, Faculty of Science, University of Technology Sydney, Sydney NSW 2007, Australia; Department of Pathology, The First Affiliated Hospital of Gannan Medical University, Ganzhou 341000, China; Department of Geriatrics, The Seventh Affiliated Hospital of Sun Yat-sen University, Shenzhen 518107, China; Faculty of Biology, Medicine and Health, The University of Manchester, Manchester M13 9PT, United Kingdom; Department of Forensic Analytical Toxicology, School of Forensic Medicine, China Medical University, Shenyang 110122, China; International Joint Research Centre on Purinergic Signalling of Sichuan Province Chengdu University of Traditional Chinese Medicine, Chengdu 611137, China; Department of Geriatrics, The Seventh Affiliated Hospital of Sun Yat-sen University, Shenzhen 518107, China; Guangdong Provincial Key Laboratory of Digestive Cancer Research, Shenzhen 518107, China; Shenzhen Key Laboratory of Chinese Medicine Active Substance Screening and Translational Research, Shenzhen 518107, China

## Abstract

Connexins, a family of transmembrane proteins, are essential for intercellular communication in the mammalian central nervous system, particularly through their assembly into gap junction channels. This review explores the central role of connexin-mediated signalling in hypothalamic regulation of nutrient metabolism and energy homeostasis, together with the function of connexins in peripheral metabolic organs. Focusing on connexins Cx43 and Cx30, we discuss their specific expression within hypothalamic neuroglial populations, including astrocytes and tanycytes, and their involvement in glucose sensing, metabolic signalling, and neuroendocrine regulation. Functional studies demonstrate that hypothalamic Cx43 is dynamically regulated by metabolic status, while its knockdown impairs glucose-stimulated insulin secretion and systemic energy balance. Tanycytes, interconnected into a Cx43-dependent network, play a critical role in relaying metabolic cues from cerebrospinal fluid and blood to hypothalamic neurons, thereby orchestrating adaptive responses to glycaemic changes. The review further details how the disruption of connexin-mediated coupling in tanycytes leads to impaired glucose sensing, altered neuronal activity, and systemic metabolic disorders. Beyond the hypothalamus, this review also summarizes how connexins in peripheral metabolic organs contribute to metabolic homeostasis, inflammatory regulation, and disease progression. Collectively, these discoveries highlight the fundamental importance of connexins in integrating metabolic signals and maintaining systemic homeostasis.

## Introduction

Connexins (Cx) belong to a family of transmembrane proteins that assemble into hexameric hemichannels (also called connexons) in the plasma membrane. There are 21 known connexin isoforms in humans [1]. Connexins are widely expressed across most mammalian cell types, including cardiomyocytes, hepatocytes, neuroglial cells, adipocytes, pancreatic β-cells, and endothelial cells. However, connexins are absent in some highly specialized or terminally differentiated cells, such as mature erythrocytes, and spermatozoa [1].

**Figure 1.**
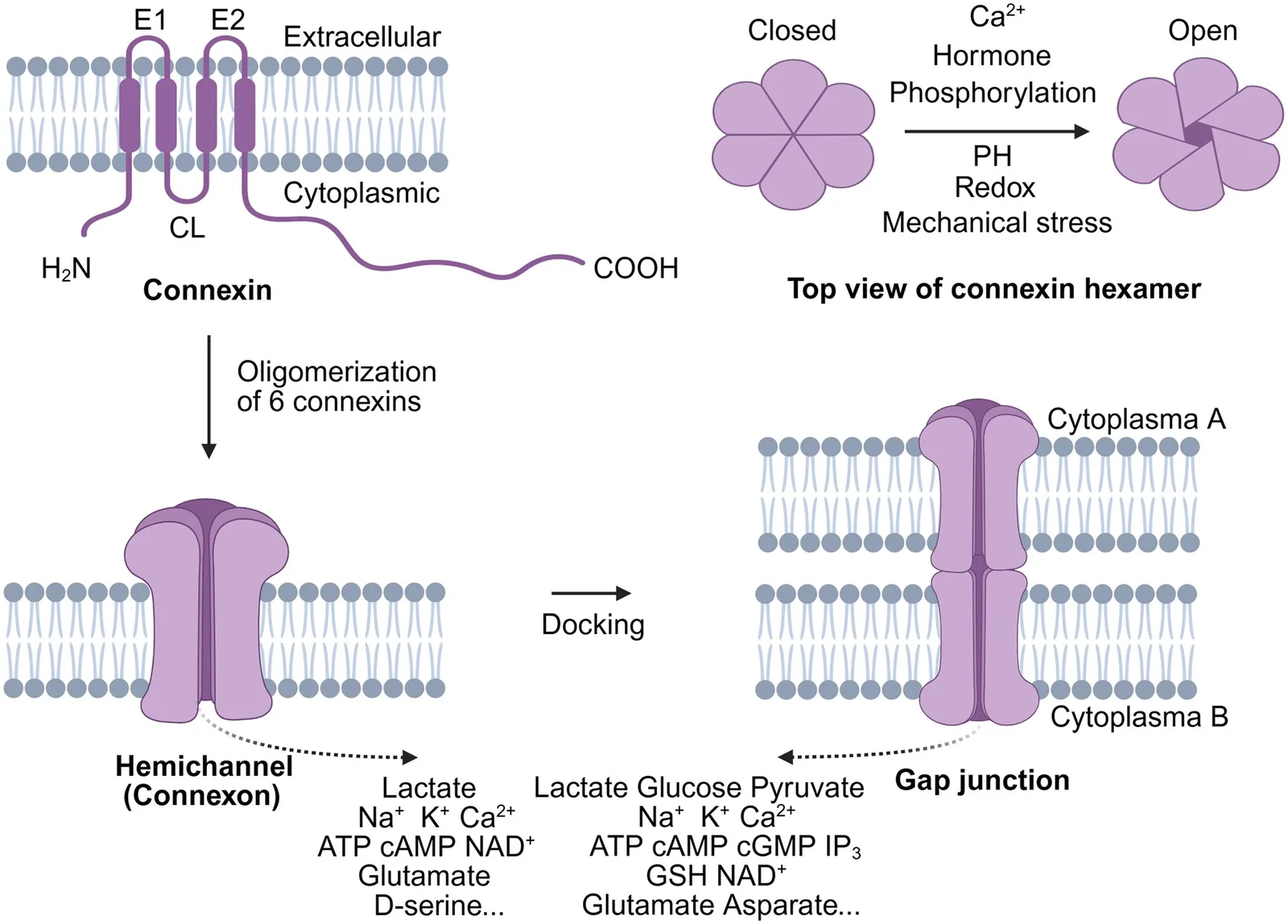
Connexin assembly into hemichannels and gap junction channels, and their permeability to small molecules. Connexin subunits form hexameric hemichannels (connexons) that dock between adjacent cells to create intercellular gap junction channels, enabling the exchange of ions and metabolites (< 1 kDa).

When connexins from adjacent cells align and dock, they form gap junction channels, enabling direct cytoplasmic exchange of ions, second messengers, and small metabolites (< 1 kDa), including ATP, glucose, lactate, NAD^+^, and cAMP, between adjacent cells (Fig. 1) [1]. Connexins are also involved in nutrient metabolism, with Cx43, Cx40, Cx30, Cx32, and Cx36 being the most studied in this context [1]. These connexins exhibit cell-type-specific expression patterns and form homo- or heteromeric channels with varying permeability and gating properties, regulated by physiological and pathological contexts. Current evidence supports the critical role of connexins, particularly those within hypothalamic neuroglia, in orchestrating nutrient sensing, neuroendocrine signalling, and systemic metabolic balance. Connexins mediate intercellular communication through both gap junctions and hemichannels, to enable intricate crosstalk between neuroglia and neurones and integrate peripheral metabolic cues to fine-tune central homeostatic responses. Disruptions in connexin function, whether in astrocytes, tanycytes, or neurones, can substantially impact glucose metabolism, feeding behaviour, and energy homeostasis, highlighting these proteins as potential targets for therapeutic intervention in metabolic disorders.

## Connexins in central regulation of nutrient metabolism

The hypothalamus acts as a central regulatory hub integrating hormonal, nutrient, and neural inputs to control energy homeostasis, glucose and lipid metabolism, and diverse neuroendocrine functions. Hypothalamus contains distinct hypothalamic nuclei, including the arcuate nucleus (ARC), paraventricular nucleus (PVN), ventromedial nucleus of the hypothalamus, dorsomedial hypothalamic nucleus, lateral hypothalamic area, and the suprachiasmatic nucleus [2–4]. Hypothalamic regulation of glucose metabolism is partly mediated by glucose-sensing neurones. Glucose-excited neurones increase their activity in response to rising extracellular glucose levels, and their activity decreases as glucose levels fall. In contrast, the activation of glucose-inhibited neurones negatively correlates with circulating glucose levels [5, 6]. However, accumulating evidence suggests that not all neurones dwelling in hypothalamic nuclei directly sense glucose. Instead, some of them rely on intercellular communication with specialized neuroglial cells, such as tanycytes, through gap junctions formed by connexins [7]. Moreover, the neuroendocrine system relies on precise coordination between tropic hormone releasing neurons and endocrine glands to maintain systemic homeostasis [8]. Connexin gap junctions enable direct intercellular communication, playing a key role in central neuroendocrine regulation processes such as tropic hormone release, stress response and reproductive function, which has been extensively reviewed elsewhere [9]. This review will focus on the hypothalamic cell populations that express connexins and discuss how alterations in connexins contribute to distinct metabolic phenotypes.

### Glial connexins in the hypothalamus

Numerous *in vivo* studies have demonstrated that astroglial connexins, particularly Cx43, play an essential role in hypothalamic glucose sensing [7, 10, 11]. Both Cx43 and Cx30 are widely expressed in the mediobasal hypothalamus. In rats, mediobasal hypothalamic Cx43 expression is dynamically regulated by metabolic status, being downregulated after 24 h of fasting and upregulated in response to acute (3 h) and chronic (48 h) hyperglycaemia [10]. Knockdown of Cx43 in the mediobasal hypothalamus by siRNA led to a marked reduction in glucose-induced insulin secretion without altering basal levels of blood glucose, insulin, or the expression of major glucose and lactate transporters in the mediobasal hypothalamus, suggesting the role of connexin-dependent neuroglial syncytium in the initiation of central glucosensory signals [10]. These findings also support the concept that neuroglial Cx43, while not acting as a glucose sensor *per se*, has its gap junctions dynamically regulated by changes in glucose availability which is indispensable for appropriate insulin responses to changes in circulating glucose availability [10].

**Figure 2.**
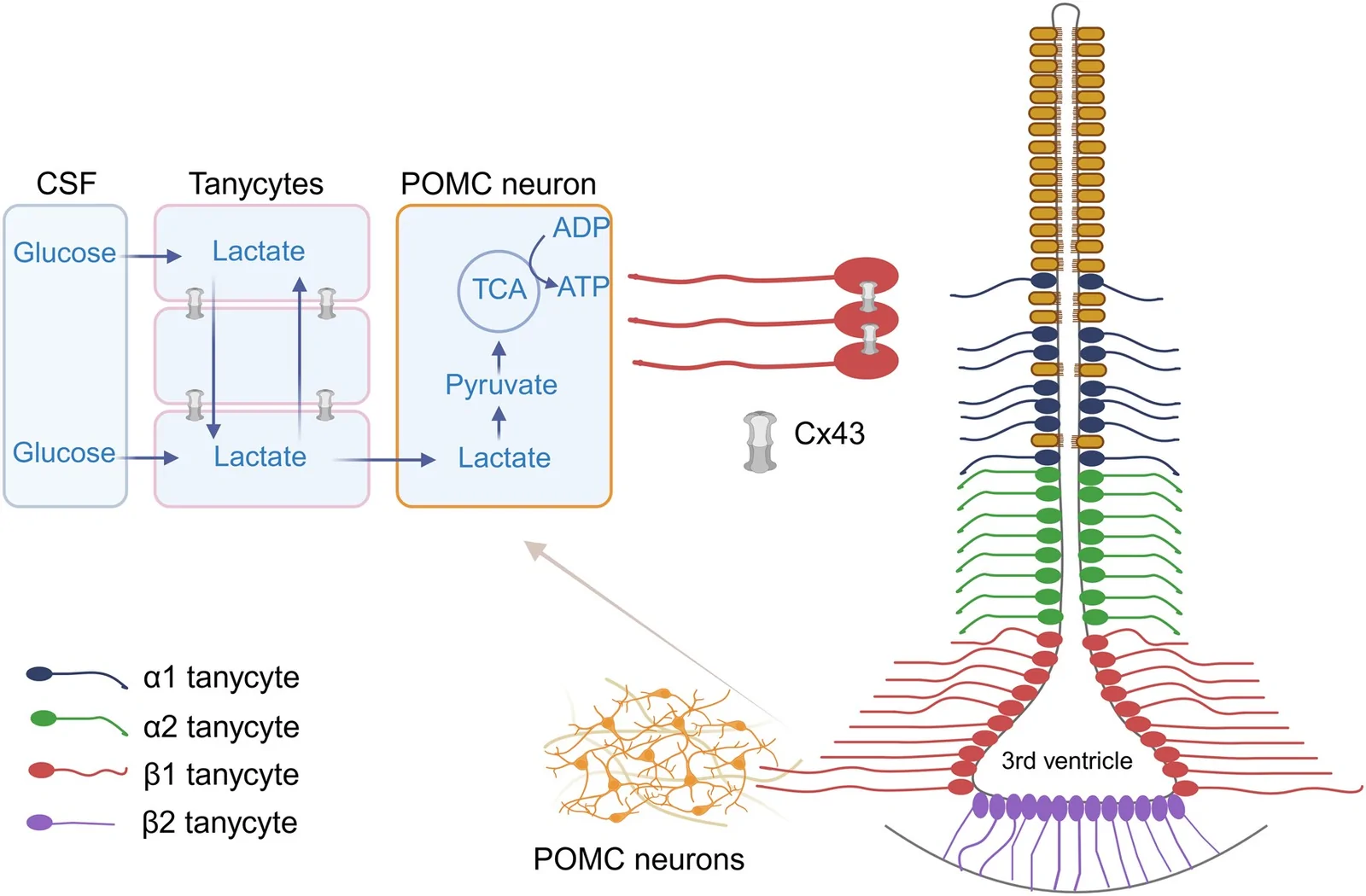
Tanycytic network assembled by connexin43 mediates lactate transport to regulat POMC neuronal activity. Connexin43-coupled tanycytes form a metabolic network that shuttles lactate from tanycytes to POMC neurones, supporting hypothalamic energy substrate sensing and neuronal firing.

Cx43 is predominantly expressed in tanycytes within the mediobasal hypothalamus and is especially enriched at the ventricular border [10]. Probing primary rat tanycyte cultures with immunohistochemical analysis confirmed that Cx43 is the predominantly expressed connexin isoform in tanycytes [11]. Tanycytes (from Greek verb τανυω, *tanyo*, which means to stretch or to elongate [12]) are a specialized population of ependymoglia with radial morphology lining the wall of the third ventricle, particularly within the mediobasal hypothalamus. They are divided into α and β subtypes based on their anatomical position and molecular markers [13, 14]. Tanycytes are in direct contact with the cerebrospinal fluid (CSF) and extend long processes projecting to hypothalamic neurones. This positions tanycytes as key sensors of nutrient metabolism needed for neuroendocrine regulation [13, 15, 16]. Tanycytes regulate peripheral glucose metabolism through their control of proopiomelanocortin (POMC) neuronal activity [7]. Tanycytes sense CSF glucose levels and metabolize glucose into lactate, which is then transferred to adjacent POMC neurones by monocarboxylate transporters. This lactate shuttle is supported by a Cx43-dependent tanycytic gap junction network, which amplifies and synchronizes the metabolic response across the tanycytic layer [7]. POMC neurones act as key hypothalamic glucose sensors, integrating nutrient-derived signals to regulate systemic energy balance [17]. The bioavailability of tanycyte-derived lactate is critical for maintaining POMC neuronal firing; disruption of lactate transport, either by knocking down monocarboxylate transporters or by deleting Cx43, leads to reduced POMC activity, and subsequently, impaired central glucose sensing ability (Fig. 2) [7]. Given the role of POMC neurones in feeding regulation, impaired melanocortin signaling results in defective appetite suppression and consequent pathological hunger [18]. Thus, central impairments of glucose sensing ultimately translate into systemic metabolic disorders, characterized by hyperphagia and related obesity, and a shift in whole-body substrate utilization from lipid to carbohydrate oxidation, as measured by an increased respiratory exchange ratio [7].

Cx43 is mainly localized to the lateral membranes of α-tanycytes, connected to key metabolic nuclei, including the ARC, dorsomedial hypothalamic nucleus (DMH), and ventromedial hypothalamic nucleus (VMH); in contrast, β-tanycytes exhibit lower levels of Cx43 expression [19]. Cx43 is also detected on the apical membranes of both α- and β-tanycytes at the ventricular surface, where gap junctions are typically absent [19]. This subcellular distribution suggests that Cx43 may form hemichannels at the apical membrane, potentially enabling direct exchange of small molecules between the CSF and tanycytic cytoplasm, thereby contributing to CSF nutrient sensing [19]. Functional studies demonstrate that β-tanycytes form a highly coupled cellular network primarily mediated by Cx43 [11]. In acute mouse hypothalamic slices [11], deletion of Cx30 alone had little effect on intercellular coupling revealed by biocytin diffusion in tanycytes; dual loss of Cx30 and Cx43, however, led to a > 90% reduction in coupling, which nearly abolished both tanycyte–tanycyte and tanycyte–parenchymal neuroglial (mainly astrocytes) communications [11]. Double knockout of Cx30 and Cx43 in tanycytes led to a more than 10-fold increase in membrane resistance of tanycytes [11], reflecting high levels of Cx43, which forms both gap junctions and hemichannels along tanycytic membranes. These observations highlight the key contribution of Cx43 to tanycytic coupling and membrane conductance. Cx43 was also observed in tanycytic endfeet contacting capillaries in the median eminence and ARC, suggesting multiple metabolic communication routes [19]. Altogether, Cx43 is a key structural and functional component of the tanycyte networks, enabling them to integrate metabolic cues from CSF and blood to control neuroendocrine output [19].

Glucose sensing by tanycytes involves a unique signalling cascade (distinct from that of pancreatic β cells) that integrates classical glucosensing elements with Cx-mediated intercellular communication. Glucose triggers intracellular Ca^2+^ signals in tanycytes in a dose-dependent manner [20, 21]. Unlike pancreatic β-cells, where glucose sensing relies on extracellular Ca^2+^ influx through voltage-dependent Ca^2+^ channels, the glucose-induced rise in intracellular Ca^2+^ in tanycytes persists in the absence of extracellular Ca^2+^ [20, 21]. This indicates a predominant role for intracellular Ca^2+^ stores in the glucosensing response. Upon glucose uptake and subsequent metabolism through glycolysis, intracellular ATP levels rise, leading to K_ATP_ channel closure and opening of Cx43 hemichannels [20, 21]. Cx43 hemichannels facilitate ATP release to the extracellular space, which in turn activates purinergic P2Y_1_ receptors, generation of inositol 1,4,5-trisphosphate (IP_3_) and Ca^2+^ release from the endoplasmic reticulum (ER), thereby sustaining the glucosensing Ca^2+^ signal [21]. Notably, the glucose-induced intracellular Ca^2+^ signals were completely abolished by pharmacological blockade of Cx43 hemichannels using La³^+^, Gap26 (a connexin43-mimetic peptide that prevents channel opening by targeting the first extracellular loop), or Cx43E2 (a specific antibody raised against the second extracellular domain of Cx43 that sterically blocks hemichannel gating) [21]. The fact that the Ca^2+^ rise is completely abolished indicates that it is not a simple IP_3_-mediated ER release. Rather, Cx43 hemichannels are required upstream, likely by releasing ATP to activate purinergic receptors, which then drives the IP_3_-dependent component. Consistently, glucose-evoked increases in non-selective membrane currents recorded under whole-cell patch-clamp can be fully reversed by Cx43E2 [21]. Together, these results demonstrate that Cx43 hemichannels are critical regulators of Ca^2+^-dependent glucose sensing in tanycytes.

Neuroglial Cx43 hemichannels were identified as active contributors to the central regulation of food intake [19, 22]. Astroglial hemichannel activity was high in acute hypothalamic slices under baseline (vehicle-treated) conditions, as measured by ethidium bromide uptake [22]. Intracerebroventricular injection of Cx43 hemichannel blocker TAT-GAP19 in mice markedly reduced food intake by about 56% and meal size by about 44% during the dark phase, without affecting meal duration [22]. TAT-GAP19 also increased the inter-meal interval by 2.8-fold, without altering blood glucose level, energy expenditure, or locomotor activity [22]. This anorexigenic effect of Cx43 inhibitors is accompanied by increased c-Fos expression in feeding-related nuclei, including the ARC, PVN, and nucleus of the solitary tract. Thus, neuroglial Cx43 hemichannels mediate a tonic orexigenic signal and are capable of shaping neuronal activity in energy-regulation circuits [22]. However, the specific cell types (astrocytes, tanycytes or both) mediating Cx43 hemichannel-dependent control of food intake remain unclear. Astrocytes are considered one of the contributors due to their high expression of Cx43 in the hypothalamus and dorsal vagal complex, and their established role in astrocyte–neuron communication [23]. However, Cx43 is also expressed in tanycytes. Given the glucose-sensing function of tanycytes, their involvement in feeding regulation cannot be excluded, which requires further investigation. It is yet to be determined whether microglia also play a role, which can be determined using cell type-specific Cx43 deletion in future studies.

### Connexins in hypothalamic neurones

Although mature neurones generally express connexins at relatively low levels, several isoforms such as Cx36, Cx45, and Cx50 are present in distinct neuronal populations and contribute to the regulation of memory and behaviours (such as learning, spatial navigation, and fear-related responses), which has been comprehensively reviewed elsewhere [24]. In the rodent hypothalamus, neuronal gap junction coupling increases from birth and peaks around postnatal day 15, then markedly declines by postnatal day 30 [25]. This developmental uncoupling is accompanied by reduced expression of Cx36 and parallels the shift from widespread electrical coupling towards more localized chemical synaptic communication. This process is driven by *N*-methyl-d-aspartate receptor-mediated Ca^2+^ influx, which activates CaMKII/PKC cascade and cAMP response element-binding protein-dependent downregulation of Cx36 [25].

While developmental studies established the temporal dynamics and regulatory mechanisms of neuronal gap junction uncoupling, much less is known about the functional relevance of neuronal connexins in the mature brain, particularly in the context of metabolic control. A role for neuronal gap junctions, specifically within hypothalamic agouti-related peptide (AgRP) expressing neurones that contribute to their regulation in energy metabolism, was uncovered recently [26]. Whole-cell patch-clamp recordings demonstrated that pharmacological inhibition of gap junctions led to membrane hyperpolarization and a 50%–70% reduction in action potential firing of AgRP-expressing neurones [26]. In mice with AgRP neurone-specific Cx43 knockout, Cx43 deletion in AgRP neurones had no significant effect on baseline energy homeostasis [26]. Even under the strong metabolic challenge of 24-hour fasting, AgRP^ΔCx43^ mice displayed normal metabolic responses, including recovery of body weight, fat and lean mass, food intake, and glycaemia (only in male mice) during subsequent refeeding, suggesting a redundant mechanism to ensure survival, such as Cx32 or Cx36, that may compensate for the loss of Cx43 in the role of feeding regulation [26]. However, only male AgRP^ΔCx43^ mice showed partial resistance to high-fat diet (HFD)–induced obesity, despite no detectable differences in energy intake or expenditure [26]. This suggests a significant sex-dependent Cx43 function in AgRP neurones. Several other factors may contribute, such as the change in nutrient digestion and absorption in the intestine due to differential microbiome populations, albeit 15 weeks of HFD feeding.

## Connexins in the peripheral metabolic organs

### Connexins in the liver

Gap junctional communication has long been recognized as a fundamental feature of hepatic physiology. From as early as the 1960s, the liver served as a model for understanding intercellular communication. Initial observations by Werner Loewenstein and Yoshinobu Kanno revealed that healthy hepatocytes maintain robust ionic exchange through specialized intercellular connections, a capacity markedly diminished in hepatocellular carcinoma [27]. In their seminal 1967 study, Revel and Karnovsky were the first to use lanthanum tracer-based electron microscopy to distinguish between morphologically similar types of intercellular junctions [28]. They demonstrated that certain junctions in the mouse heart and liver, although exhibiting a pentalaminar appearance typically associated with tight junctions, were in fact permeable to lanthanum and possessed an intermembrane gap of around 20 Å. In contrast, true tight junctions prevent lanthanum passage, reflecting their role as diffusion barriers. The lanthanum-permeable junctions also revealed a characteristic hexagonal array of subunits, later recognized as a structural hallmark of gap junctions. This study thus provided the first ultrastructural evidence differentiating gap junctions from tight junctions based on both tracer permeability and subunit organization [28]. In 1974, Daniel Goodenough isolated two gap junctional channel proteins from mouse liver and named them “*connexins*” [29].

The liver is a central metabolic hub that integrates nutrient sensing, energy storage, detoxification, and systemic homeostasis. In the liver, hepatocytes predominantly express Cx32, accounting for about 90% of total hepatic connexin content, with a minor contribution from Cx26, which represents about 5% of total connexins. In contrast, hepatic stellate cells and Kupffer cells, the resident macrophages of the liver, predominantly express Cx43, whereas vascular endothelial cells mainly express Cx37 and Cx40 [30–36]. Although various liver cell types express connexins, functional gap junctions are mainly observed in hepatocytes and stellate cells. In hepatocytes, gap junctions (composed of Cx32 and Cx26) occupy about 3% of the plasma membrane surface and are typically organized into discrete plaques. Each plaque, with diameters ranging from 0.2 to 1 μm, contains 10–10,000 individual channels. These channels are about 15 Å in diameter and 180 Å in length, forming direct cytoplasmic bridges that enable efficient intercellular communication [37].

**Figure 3.**
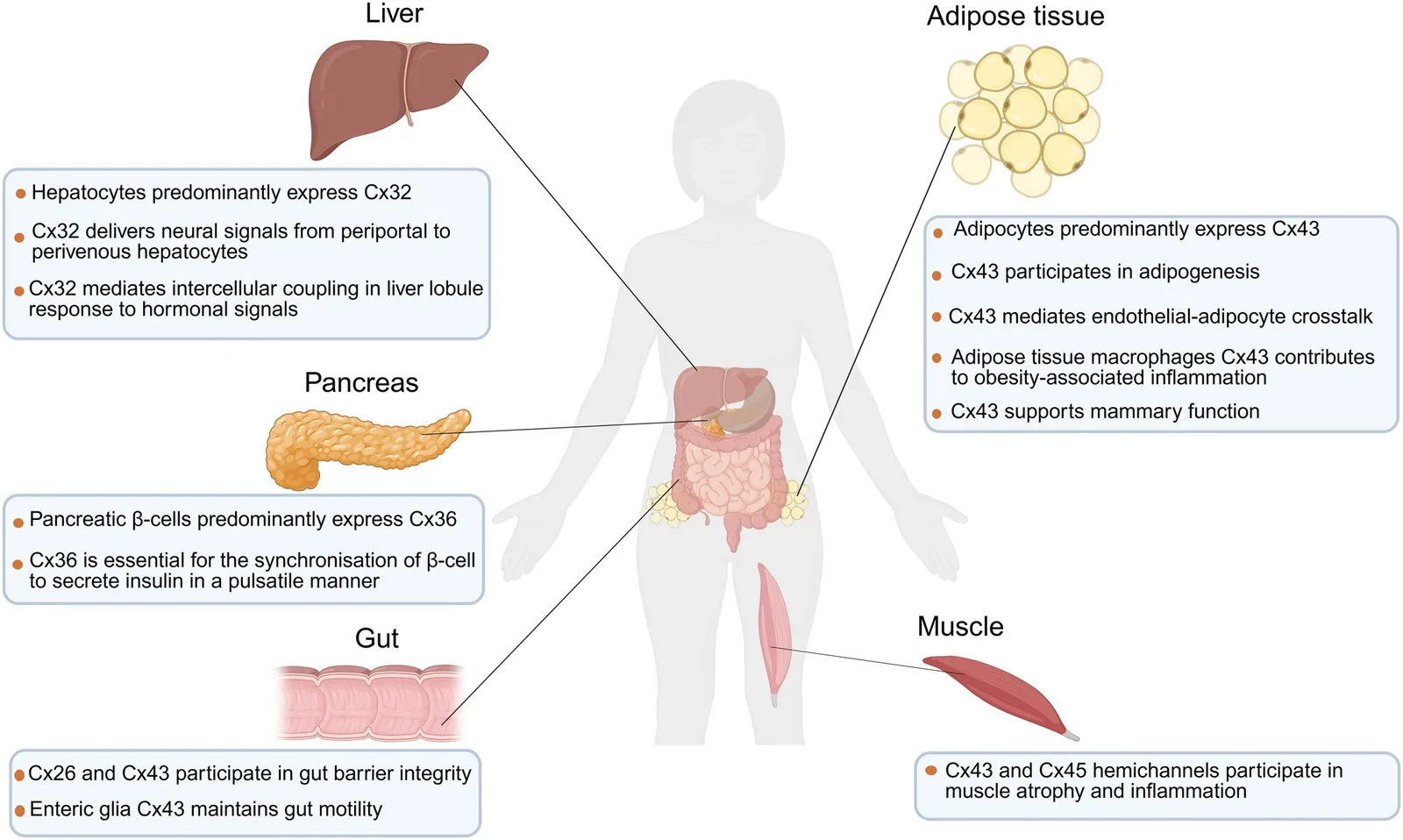
Connexins in peripheral metabolic organs. Schematic illustration showing the distribution and functional roles of connexins in peripheral metabolic organs, including liver, adipose tissue, pancreas, gut, and muscle.

Under physiological conditions, gap junctions mediate the transmission of neural and hormonal signals from periportal to perivenous hepatocytes. In both mice and rats, hepatic innervation is predominantly confined to the portal triads and typically does not extend into the hepatic lobules [38, 39]. Intralobular innervation is either absent or extremely sparse, with no direct contact observed between nerve terminals and hepatocytes [38, 39]. In Cx32-deficient mice, electrical stimulation of sympathetic nerves triggered a about 78% reduction in glucose release from glycogen stores, indicating that Cx32-containing gap junctions are critical for delivering noradrenaline signals from periportal to perivenous hepatocytes [40]. In addition to neural signals, hormonal responses are also modulated by gap junction communication. In an *ex vivo* setting, when perfused with saturating concentrations of noradrenaline or glucagon, glucose release was comparable between livers from wild-type and Cx32-deficient mice [41]. However, when hormone levels were halved, glucose release was significantly reduced in the livers from Cx32-deficient mice, indicating that Cx32 mediates intercellular coupling in liver lobule response to hormonal signals [41]. Taken together, these insights highlight the key role of connexins in guiding intercellular communication and coordinating metabolic processes across diverse cell types in the liver (Fig. 3).

## Connexins in adipose tissue

Adipose tissue derives from mesodermal mesenchyme and contains morphologically and functionally diverse adipocytes. Connexins have emerged as regulators of adipose tissue development and function. Cx43 is the predominant connexin isoform in both mouse subcutaneous and mesenteric white adipose tissue (WAT), as well as in 3T3-L1 preadipocytes [42]. In contrast, Cx37, Cx40, and Cx45 were detected at much lower levels, and other isoforms such as Cx26, Cx30, Cx32, Cx36, and Cx47 are absent [42]. Similar to WAT, there are also high levels of Cx43 in the brown adipose tissue (BAT) [43]. In mouse BAT, several other connexin isoforms, including Cx45, Cx59, Cx37, and Cx31.1 were found. Cx43 level is much higher in BAT than in WAT [43]. In rats, both the number and area of gap junctions in BAT are increased after birth, reaching a peak during the thermogenically active phase (birth to 3–4 weeks), and decline thereafter. In BAT, the gap junction area to cell volume ratio is closely associated with thermogenic activity, suggesting a role of intercellular coupling in thermogenesis [44]. Notably, Cx43 plays an active role in promoting the beiging of WAT [45] (Fig. 3).

Recent evidence highlighted a key role for Cx43 in regulating systemic energy homeostasis through mesenchymal lineage cells, which differentiate into adipocytes (Fig. 3). Conditional deletion of *Gja1*, the gene encoding Cx43, in mesenchymal precursors using a Dermo1-Cre driver resulted in reduced adiposity and partial resistance to high-fat diet–induced weight gain [46]. These Cx43-deficient mice exhibited enhanced energy expenditure, increased locomotor activity, improved glucose tolerance, and greater BAT thermogenic activity [46]. Mechanistically, the protective metabolic phenotype appears to arise from developmental reprogramming of adipose precursors rather than from changes in mature adipocytes. The loss of Cx43 during the early adipogenic stage leads to long-term alterations in WAT and BAT function. In WAT, *Gja1* ablation enhances adipogenic gene (*Cebpa*, *Pparg*, and *Adipoq*) expression and glucose uptake by Glut4, suggesting improved glucose utilization. In BAT, *Gja1* ablation prevents whitening and promotes thermogenesis and lipolysis upon HFD eating and cold exposure, associated with increased expression of thermogenic and lipolytic genes [46]. The loss of Cx43 during the early differentiation stage leads to a reprogramming of adipose precursors [46]. These transcriptional changes emerge during early stages of adipogenesis and are not observed when Cx43 is deleted in mature adipocytes, suggesting that the protective metabolic phenotype originates from alterations in lineage commitment and differentiation, rather than from mature adipocyte function [46].

Cx43 mediates endothelial–adipocyte communication in adipose tissue (Fig. 3). Single-cell transcriptomics identified Cx43 as the only connexin abundantly expressed in both capillary endothelial cells and adipocytes [47]. Gap junctions between these cells were demonstrated by fluorescence recovery after photobleaching-based dye transfer assays, which were significantly impaired by Cx43 inhibition or knockdown [47]. Although Cx43 expression is increased in capillary endothelial cells by HFD consumption, this is accompanied by enhanced phosphorylation at serine 368, a post-translational modification known to close gap junction channels [47]. This suggests a maladaptive response: while endothelial cells may attempt to upregulate Cx43 to sustain intercellular communication under lipid stress, the concomitant phosphorylation disrupts gap junctional coupling, thereby impairing endothelial–adipocyte metabolic coordination. Under HFD feeding, endothelial-specific Cx43 knockout mice displayed a more pronounced increase in body weight, inguinal WAT expansion, and serum lipid levels (total cholesterol and triglycerides) compared to control mice [47]. Conversely, transgenic mice overexpressing a non-phosphorylatable, constitutively open Cx43 channel (Cx43 S368A) showed reduced fat mass and lower circulating lipid levels [47]. However, manipulations of Cx43 do not affect glucose tolerance or insulin sensitivity, suggesting that Cx43 in the fat tissue primarily regulates lipid metabolism rather than glucose homeostasis [47].

Adipose tissue macrophages are key mediators of obesity-induced systemic low-grade inflammation. In F4/80^+^ adipose tissue macrophages, Cx43 is upregulated under high-fat diet conditions [48]. In a tamoxifen-inducible macrophage-specific Cx43 knockout model (Csf1r-CreER; Gja1^flox/flox), inflammatory cytokine production and inflammasome activation were reduced. Of note, Cx43-deficient mice showed improved glucose tolerance and insulin sensitivity, suggesting that macrophage Cx43 contributes to obesity-associated metabolic malfunction due to increased macrophage induced adipose tissue inflammation [48] (Fig. 3).

Moreover, Cx43 is also the most abundantly expressed connexin in mammary adipose tissue [49]; expression of Cx43 increases during lactation, suggesting a potential role in supporting mammary function. It is decreased in obesity, a condition in which mammary functions, particularly adipocyte remodelling, milk production, and the maintenance of breastmilk quality, are impaired [49]. In doxycycline-inducible, adipocyte-specific Cx43 knockout mice (Apn-Cx43-KO), mammary adipocyte remodelling was affected during lactation, resulting in reduced breastmilk lactose concentration, and growth restriction in cross-fostered neonates [49]. These findings highlight a previously unrecognized role of adipocyte Cx43 gap junctions in modulating breastmilk quality, especially under metabolic stress [49] (Fig. 3).

## Connexins in the pancreas

Cx36 is the primary and most abundant connexin isoform in pancreatic β-cells, where it forms gap junctions that synchronize electrical activity and Ca^2+^ signalling, essential for glucose-stimulated insulin secretion [50]. It has little to no expression in other endocrine cell types [51]. Consistently, in lacZ reporter knock-in mice, Cx36 expression is restricted to β-cells, whereas Cx43 and Cx45 are confined to islet endothelial and vascular smooth muscle cells, respectively [52]. Exposure of β-cells and primary rat pancreatic islets to a solution with a high concentration of glucose leads to a dose- and time-dependent reduction in Cx36 mRNA and protein levels [53], which was reversible after the restoration of ambient glucose level. Mechanistically, glucose-induced downregulation of Cx36 is mediated by the cAMP–PKA signalling pathway and thus can be completely inhibited by the PKA inhibitor H89 [53]. Knockdown of Cx36 in mice abolished gap junctions in β-cells, while preserving islet architecture [54]. Functionally, Cx36-deficient islets completely lose synchronized glucose-induced intracellular Ca^2+^ oscillations and fail to secrete insulin in a pulsatile manner, despite maintaining overall insulin output in response to glucose [54]. The Cx36-null islets exhibit elevated basal insulin secretion under low-glucose conditions [54]. Thus, Cx36-mediated gap junction coupling is essential for the synchronization of β-cell intracellular Ca^2+^ dynamics and glucose-regulated baseline and pulsatile insulin release [54] (Fig. 3).

## Connexins in skeletal muscle

Skeletal muscles account for about 40% of total body mass in healthy adults [55]. These muscles play a major role in postprandial glucose utilization, responsible for about 80% of insulin-stimulated whole-body glucose uptake [56]. Despite a relatively modest resting metabolic rate per unit, skeletal muscle contributes around 30% of resting energy expenditure due to its large volume [57]. During exercise, skeletal muscle ATP turnover can increase 100-fold above rest, highlighting its metabolic flexibility [58]. While connexin gap junctions are well-established in cardiac muscle, where they enable synchronized myocardial contraction [59–61], their presence in skeletal muscle is more restricted. Only Cx39, Cx40, Cx43, and Cx45 were found in developing myoblasts and injured adult skeletal muscle. In contrast, connexin expression is absent in healthy adult skeletal muscle [62].

A recent study revealed a critical role of connexin hemichannels in denervation-induced skeletal muscle atrophy [63]. In denervated fast-twitch myofibers, Cx39, Cx43, and Cx45 hemichannels are activated, which mediates ATP release and increases sarcolemmal permeability to small molecules. ATP, in turn, activates P2X_7_ receptors and induces a feed-forward loop that promotes Ca^2+^ influx, NF-κB activation, and inflammasome signalling [63]. Genetic ablation of Cx43 and Cx45 in skeletal muscle markedly reduced inflammatory responses and preserved muscle fibre cross-sectional area following denervation, highlighting connexin hemichannels as upstream regulators of sterile inflammation and key drivers of muscle wasting [63]. These findings suggest connexin hemichannels as promising therapeutic targets for mitigating muscle atrophy in neuromuscular disorders (Fig. 3) [63].

Diabetic myopathy is characterized by a significant reduction in the cross-sectional area of tibialis anterior myofibers and an increase in sarcolemmal permeability. In the sarcolemma, connexin expression is elevated, particularly Cx39, Cx43, and Cx45, along with upregulated P2X_7_ receptor [64]. Boldine, a natural alkaloid known to inhibit Cx43/45 hemichannels and P2X_7_ receptors, effectively prevented increased permeability and muscle atrophy in diabetic rats [64]. These findings suggest that modulation of connexin hemichannels may hold therapeutic potential not only in diabetic muscle degeneration but also in other conditions characterized by sarcolemmal dysfunction, such as denervation-induced atrophy and sepsis-related muscle wasting.

## Connexins in the gut barrier

The bidirectional interaction between the gut microbiota and host metabolism is increasingly recognized as a key determinant of metabolic health. The conserved gap junction protein Cx26, encoded by *Gjb2* gene, is expressed in epidermal and intestinal epithelia, where it contributes to the maintenance of physical and osmotic barriers against environmental stressors (Fig. 3) [65]. The risk of loss-of-function mutations in *Gjb2* is high in humans [66]. Although biallelic mutations cause congenital deafness, the presence of heterozygous carriers raises the possibility of a selective advantage linked to enhanced epithelial barrier function [65, 66]. Notably, *Gjb2* variant carriers have lower susceptibility to gastrointestinal infections, particularly diarrheal diseases caused by enteropathogenic *Escherichia coli* and *Shigella flexneri* [65]. This may reflect an adaptive remodelling of the epithelial landscape, including increased thickness of the intestinal epithelium, which is in some respects analogous to the epidermal barrier of the skin [66], which creates a suboptimal microenvironment for microbial colonization. As such, the preservation of *Gjb2* mutations in certain populations may be driven by positive selection for barrier resilience in pathogen-rich environments [65]. In addition, Cx43 has emerged as a key regulator of intestinal epithelial barrier function (Fig. 3). Activation of Toll-like receptor 2 (TLR2) enhances Cx43 transcription and membrane localization, promoting epithelial repair, while TLR2 mutations impair Cx43 stability and gap junctional communication, exacerbating inflammation [67]. However, direct links between connexin-regulated barrier function, microbiota modulation, and systemic metabolism remain poorly understood and warrant further investigation.

## Connexin in enteric glia

Enteric glial cells are the principal non-neuronal cells of the enteric nervous system, sharing many morphological and functional features with astrocytes [68]. They express similar molecular markers to astroglia, including GFAP, vimentin, S100β, and Cx43 [68]. In the enteric nervous system, enteric glial cells generate intercellular Ca^2+^ waves through Cx43 hemichannels, which contribute to the release of ATP into the extracellular space to activate purinergic receptors on neighbouring glia, thereby sustaining the propagation of Ca^2+^ waves essential for normal gut motility (Fig. 3) [69]. Pharmacological blockade of Cx43 using carbenoxolone or the selective mimetic peptide Gap26, as well as enteric glia-specific deletion of Cx43 in mice, markedly reduced purinergic Ca^2+^ signalling, impaired excitatory and inhibitory neuromuscular transmission, delayed colonic transit, and altered faecal composition [69]. Notably, these Cx43-dependent Ca^2+^ responses decline with age, suggesting that dysregulation of glial Ca^2+^ signalling contributes to the age-related slowing of gastrointestinal transit [69].

## Connexins in metabolic diseases

### Diabetes mellitus

Dysregulation of connexins in diabetes disrupts cell-to-cell communications and exacerbates insulin defects and tissue damage in the pancreas, retina, kidney, and vasculature. Particularly, Cx36 and Cx43 are involved in the pathogenesis of diabetic complications, such as retinopathy, nephropathy, and cardiovascular malfunction. These aspects will be discussed in detail in the following sections.

**Figure 4.**
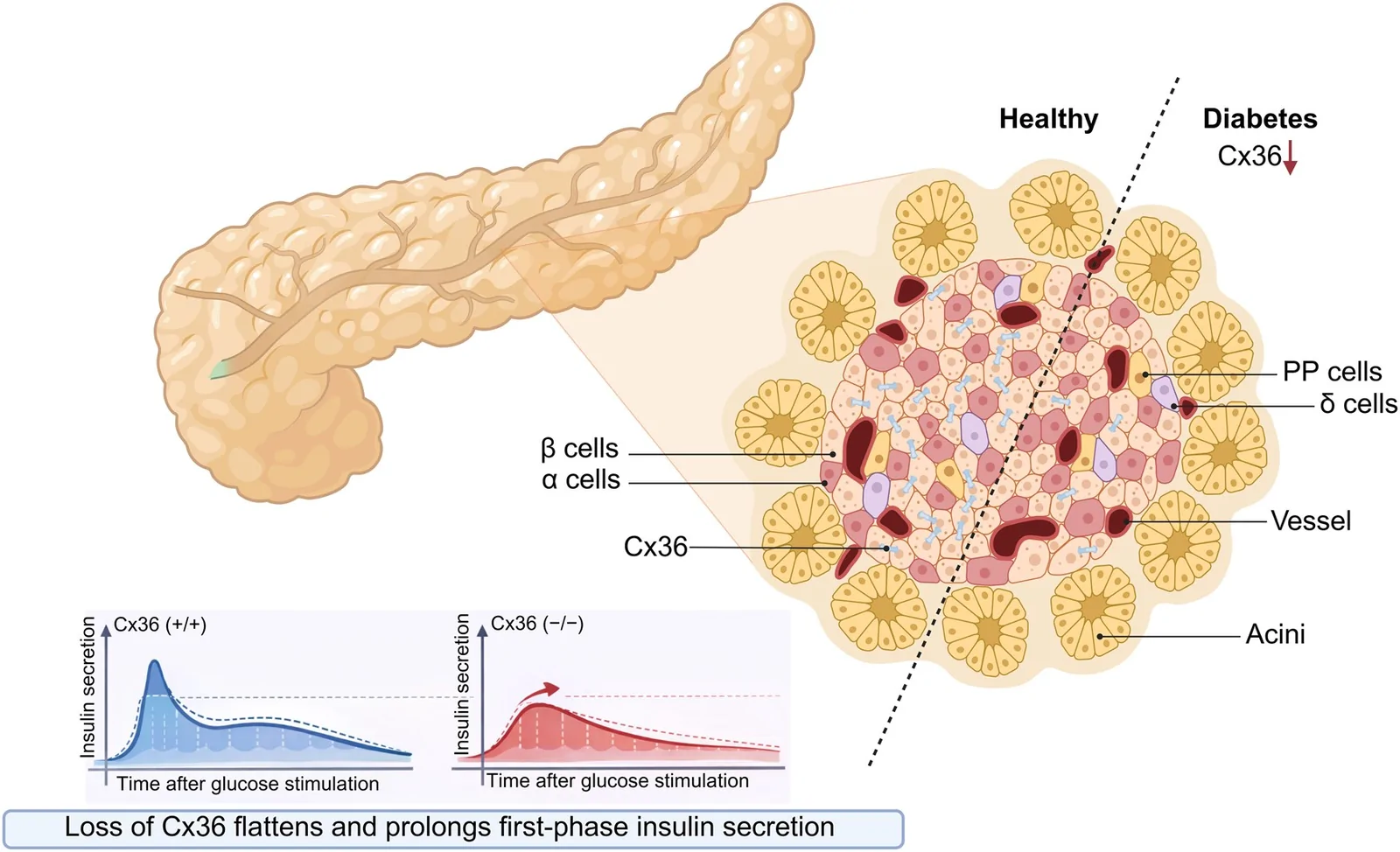
Connexin36-mediated β-cell coupling shapes insulin secretory dynamics in pancreatic islets. Schematic illustration of the pancreas and a magnified view of a pancreatic islet, highlighting the spatial organization of endocrine cell types, including β cells, α cells, δ cells, and PP cells (also known as pancreatic polypeptide cells), in close association with the islet vasculature and surrounding exocrine acini. Under healthy conditions, β cells are electrically coupled via Cx36 gap junctions, enabling synchronized Ca^2+^ activity and coordinated insulin secretion. In diabetes, reduced Cx36 expression disrupts β-cell coupling, leading to desynchronized β-cell activity. This results in a characteristic alteration of insulin secretion dynamics, with a reduced peak amplitude and prolonged first-phase insulin release following glucose stimulation. Representative insulin secretion profiles illustrate the flattening and temporal spreading of first-phase insulin secretion in the absence of Cx36, despite relatively preserved overall insulin output.

Cx36 encoding gene *Gjd2* is located at chromosome 15q14, which is a region identified as a susceptibility locus for type 2 diabetes and related metabolic disorders [70]. Cx36 expression is significantly reduced in pancreatic β-cells, leading to asynchronous insulin release and impaired glucose homeostasis [50]. In islets from both human type 2 diabetic patients and db/db mice, Cx36 gap junction coupling is significantly impaired, leading to disrupted Ca^2+^ oscillations and diminished β-cell coordination [71]. Islets from 4-week-old db/db mice, prior to overt hyperglycaemia, already displayed reduced gap junction communication and desynchronized Ca^2+^ dynamics, indicating early Cx36 malfunction during the prediabetic stage [71]. Restoration of Cx36 function through β-cell–specific Cx36 overexpression, modafinil, or a mimetic peptide (S293, targeting the Ser293 regulatory site on the C-terminal tail of Cx36) rescued gap junction function, restored Ca^2+^ synchronization, reduced cytokine-induced β-cell apoptosis, and improved glucose-stimulated insulin secretion [71]. In Cx36 knockout (Cx36^−^^/^^−^) mice, although basal insulin levels and insulin sensitivity remain unchanged, the loss of β-cell electrical coupling leads to a marked reduction in both the amplitude of first-phase insulin release and the mass of second-phase insulin pulses. These defects result in impaired glucose tolerance in mice (Fig. 4) [72]. Altered insulin secretory dynamics resemble early abnormalities observed in type 2 diabetes, suggesting that disrupted Cx36 gap junction function may contribute to disease onset by impairing islet synchronization and glucose-induced insulin release [72]. Notably, the S293 peptide restored islet function in type 2 diabetic human donors, supporting Cx36 as a promising therapeutic target for preserving islet function in diabetes [71].

In type 1 diabetes, Cx36 also exerts a protective role against β-cell injury [73, 74]. The loss of Cx36 significantly increased β-cell susceptibility to apoptosis induced by a cytokine cocktail of interleukin (IL)-1β, tumor necrosis factor-α (TNF-α), and interferon (IFN)-γ, suggesting Cx36 as a critical regulator of β-cell integrity in type 1 diabetes. The protective role was attributed to its maintenance of gap junctional communication and buffering of cytotoxic Ca^2+^ signals [73, 74]. Furthermore, proinflammatory cytokines were shown to downregulate *Gjd2* transcription by inhibiting cAMP response element-binding protein activity, suggesting that Cx36 is a key step in the progression of β-cell injury [73]. In Cx36-deficient INS-1E cells, exposure to a pro-inflammatory cytokine cocktail led to exacerbated oxidative stress, ER stress, and mitochondrial dysfunction, with increased apoptosis [74]. On the other hand, MIN6 cells overexpressing Cx36 are resistant to β-cell targeting toxins, such as streptozotocin (STZ) or alloxan [74]. Cytokine-induced downregulation of Cx36 involves the inhibition of cAMP response element-binding protein and activation of AMPK via a Ca^2+^/CaMKKβ-dependent pathway [74]. Together, these findings indicate that Cx36 protects β-cells from both cytokine-induced and chemically induced damage by preserving gap junctional communication and limiting intracellular stress responses [74].

### Diabetic microvascular complications

Vascular complications are the leading cause of morbidity and mortality in diabetes, affecting both large and small vessels. These complications are often accompanied by structural and functional alterations of the vascular wall, including changes in intercellular communication [75]. In healthy vessels, connexin gap junctions exhibit a well-defined spatial distribution, which plays a critical role in maintaining vascular homeostasis. Cx37, Cx40, Cx43, and Cx45 are the most prominent connexins expressed in vascular tissues [76]. Their distribution is heterogeneous, varying with vascular bed, vessel type, and species. Typically, Cx37 and Cx40 are co-expressed in endothelial cells, while Cx43 and Cx45 are predominantly found in smooth muscle cells [76, 77]. Cx43 is additionally present in endothelial cells at arterial branch points [78]. The pathological roles of connexins, particularly in diabetic vasculopathy, will be discussed in the following sections.

#### Diabetic retinopathy

**Figure 5.**
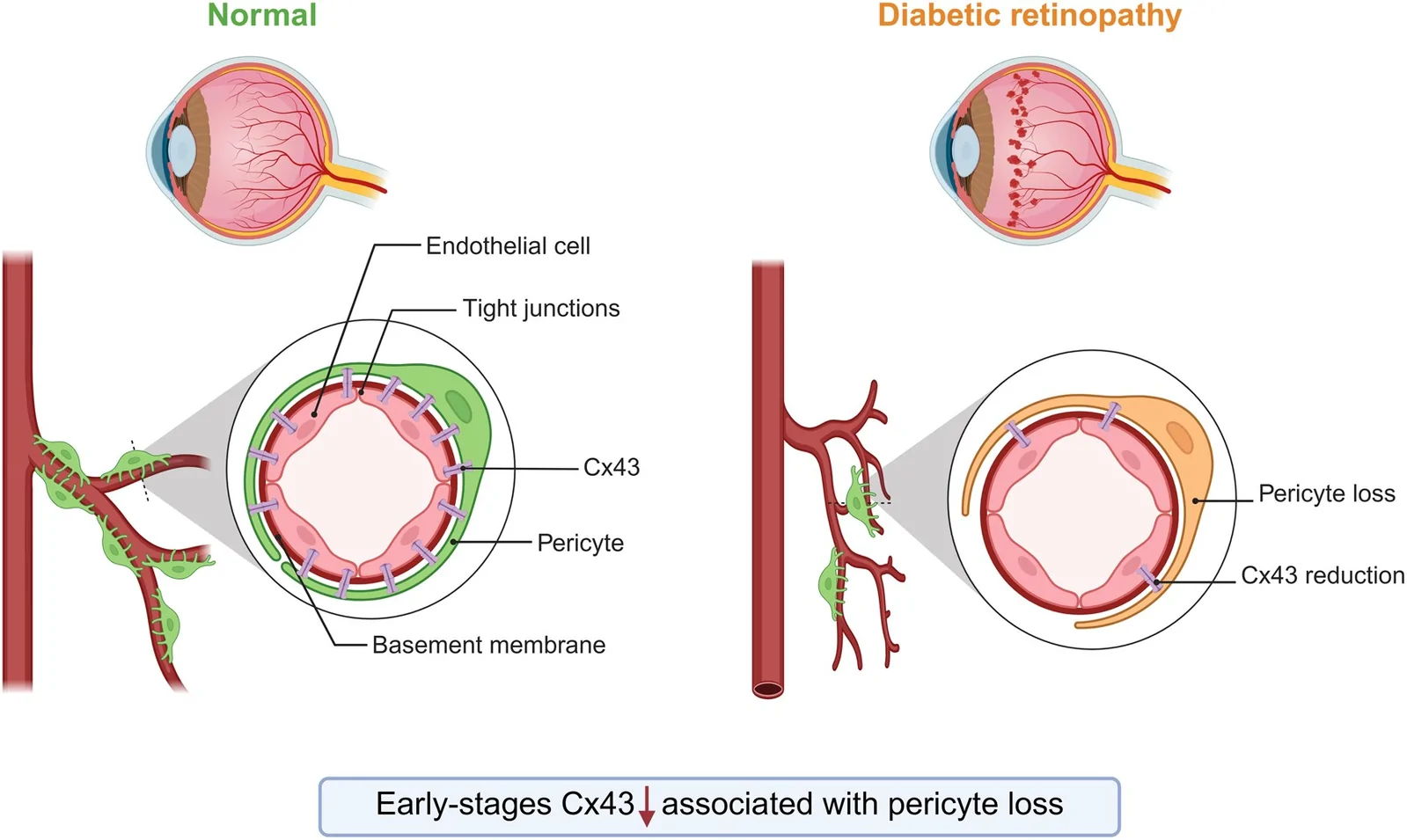
Connexin43 in pathogenesis of diabetes retinopathy. Reduced Cx43 expression and plaque density in early diabetic retinas correlate with acellular capillaries and pericyte loss.

Cx43 is the predominant connexin in the retina, including vascular endothelial cells, pericytes, and Müller glial cells, where it plays a critical role in maintaining blood–retinal barrier integrity and ensuring vascular homeostasis [79]. The pathological processes of diabetic retinopathy, including early pericyte loss and vascular endothelial growth factor (VEGF)-centred neovascularization [80, 81], are tightly linked to connexin signalling. Gap junctions enable communication between endothelial cells and pericytes [82]. Gap junction-mediated intercellular coupling was markedly diminished within days of the onset of STZ-induced diabetes, reflecting a rapid impairment in cell-to-cell communication. This reduction is not due to a direct toxic effect of STZ, since coupling remained intact in insulin-treated diabetic rats despite prior STZ exposure, suggesting the effects are glucose/insulin dependent [83]. Furthermore, the activation of protein kinase C (PKC), similar to that observed in diabetic retina, significantly suppressed gap junction function, suggesting the involvement of PKC signalling [83]. In human early-stage diabetic retinas, Cx43 protein levels are reduced by 19%, with a 38% reduction in Cx43 plaques per unit vessel length, which is strongly associated with increased numbers of acellular capillaries and pericyte loss (Fig. 5) [84]. At later stages, however, Cx43 becomes upregulated in endothelial cells, where it promotes pathological angiogenesis and inflammation. Cx43 can be upregulated in human retinal endotheliocytes in a dose-dependent manner under high-glucose conditions, promoting tube formation, reactive oxygen species production, and the secretion of Vascular Endothelial Growth Factor A (VEGFA), TNF-α, IL-1β, and intercellular adhesion molecule-1 [85]. As such, Cx43 knockdown significantly suppresses such pro-angiogenic and pro-inflammatory effects [85]. Intravitreal injection of Cx43-shRNA has also been shown to reduce retinal VEGFA expression and ameliorate retinal structural damage [85], suggestive of a target for intervention.

#### Diabetic nephropathy

Although chronic hyperglycaemia underlies both diabetic retinopathy and nephropathy, only about one-third of patients with diabetes develop chronic kidney disease [86, 87]. Diabetic nephropathy is characterized by progressive structural changes beginning with glomerular hyperfiltration, endothelial dysfunction and early inflammatory responses, followed by thickening of the glomerular basement membrane, mesangial matrix expansion, and podocyte loss [88, 89]. As the disease progresses, nodular glomerulosclerosis, most notably the formation of Kimmelstiel–Wilson nodules, emerges as a hallmark lesion. These glomerular changes are often accompanied by arteriolar hyalinosis and ultimately lead to glomerular sclerosis and nephron loss [88, 89]. In the late stages, tubulointerstitial fibrosis becomes prominent, contributing to a further decline in renal function and progression to end-stage renal disease [88, 89].

**Figure 6.**
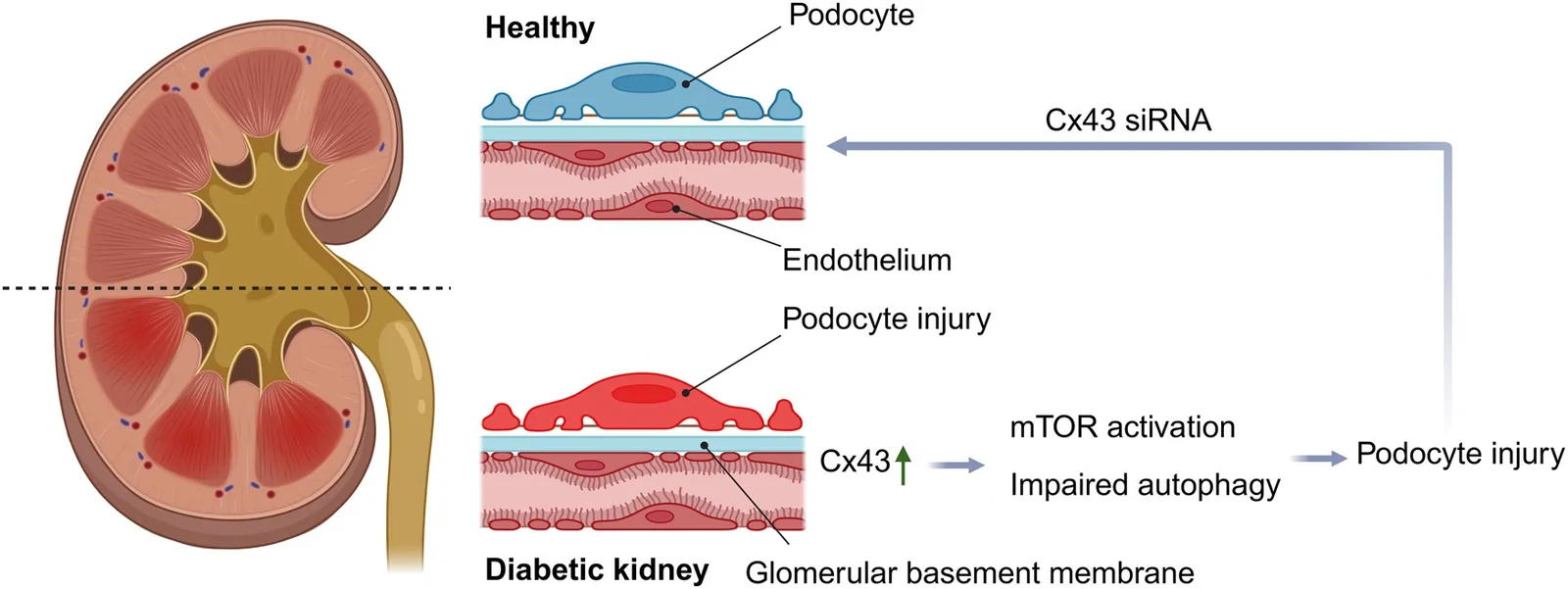
Connexin43-mediated dysregulation of autophagy contributes to podocyte injury in diabetic nephropathy. Schematic illustration of the glomerular filtration barrier under healthy and diabetic conditions. In diabetic nephropathy, Cx43 expression is upregulated in podocytes, leading to mTOR activation, impaired autophagic flux, and podocyte injury, thereby disrupting glomerular integrity. Suppression of Cx43 by siRNA restores autophagic flux and alleviates podocyte injury, highlighting Cx43 as a potential therapeutic target in early diabetic nephropathy.

In glomerular injury, Cx43 expression is elevated; for example, in IgA nephropathy, C3 glomerulopathy, and nephrotoxic serum-induced glomerulonephritis, *de novo* Cx43 appears in damaged podocytes [90]. Cx43^+^/^−^ mice show reduced proteinuria, creatinine, inflammation, and fibrosis compared to wild-type controls [90]. Moreover, Cx43-antisense treatment in mice with nephrotoxic serum-induced glomerulonephritis can preserve renal function and structure [90]. *In vitro*, Cx43 blockage with Gap26 can attenuate TGF-β–induced podocyte dedifferentiation and apoptosis [90]. In STZ-induced diabetic rats, Cx43 expression was upregulated in association with podocyte injury. Mechanistically, podocyte injury was driven by impaired autophagy, and Cx43 siRNA treatment restored autophagic flux through suppression of mTOR signalling, thereby improving kidney function (Fig. 6) [91]. These findings suggest that Cx43 overexpression in podocytes directly contributes to glomerular injury. Since podocyte damage is an early and critical event in diabetic nephropathy, suppressing Cx43 in the early stage of diabetic nephropathy may offer a promising strategy to preserve renal function.

#### Diabetic neuropathy

Diabetic neuropathy is one of the most common and devastating complications of diabetes mellitus, affecting up to 50% of patients [92]. It includes a spectrum of syndromes resulting from damage to peripheral sensory nerves, which underlie the development of sensory loss and neuropathic pain, as well as autonomic nerves, with distal symmetric polyneuropathy being the most prevalent form [92]. Characterized by sensory loss or neuropathic pain, diabetic neuropathy significantly impairs quality of life and increases morbidity [92, 93]. In diabetic neuropathy, Schwann cells are vulnerable to chronic hyperglycaemia, and demyelination is frequently observed in more advanced stages of the disease [92]. Connexins, particularly Cx32, are highly expressed in non-compact myelin regions of Schwann cells, where they form gap junction channels essential for radial diffusion of ions and small molecules across the myelin sheath [94, 95]. Research on connexins in diabetic neuropathy remains limited.

Diabetic neuropathy is a major contributor to chronic neuropathic pain. In a recent review, we comprehensively discussed the pathological roles of connexins in the development of neuropathic pain [96], underscoring their potential involvement in diabetic neuropathy–associated pain and pointing to connexins as promising therapeutic targets.

### Diabetic macrovascular complications

Diabetes mellitus is recognized as an independent risk factor for the development of cardiovascular diseases. Epidemiological evidence indicates that individuals with type 2 diabetes exhibit a 2- to 3-fold higher incidence of coronary artery disease [97] and a 2- to 5-fold increased risk of ischemic stroke compared to non-diabetic populations [98, 99]. Atherosclerosis is the core pathology behind these complications. In diabetic patients, the progression of atherosclerotic plaque is accelerated, even when low-density lipoprotein cholesterol levels are comparable to non-diabetic individuals [100], suggesting that additional mechanisms. The genetic susceptibility of macrovascular complications in diabetes mellitus includes *GLUL*, *MGMT*, *CDKN2B-AS1*, which have been summarized elsewhere [101]. Increasing evidence indicates that connexins are closely involved in the development and progression of diabetes associated atherosclerosis [102]. High glucose conditions impair gap junctional connectivity in vascular smooth muscle cells by promoting PKC-mediated hyperphosphorylation of Cx43, a mechanism that may underlie exacerbated diabetic macrovascular complications [103].

### Metabolic dysfunction–associated fatty liver disease (MASLD)

**Figure 7.**
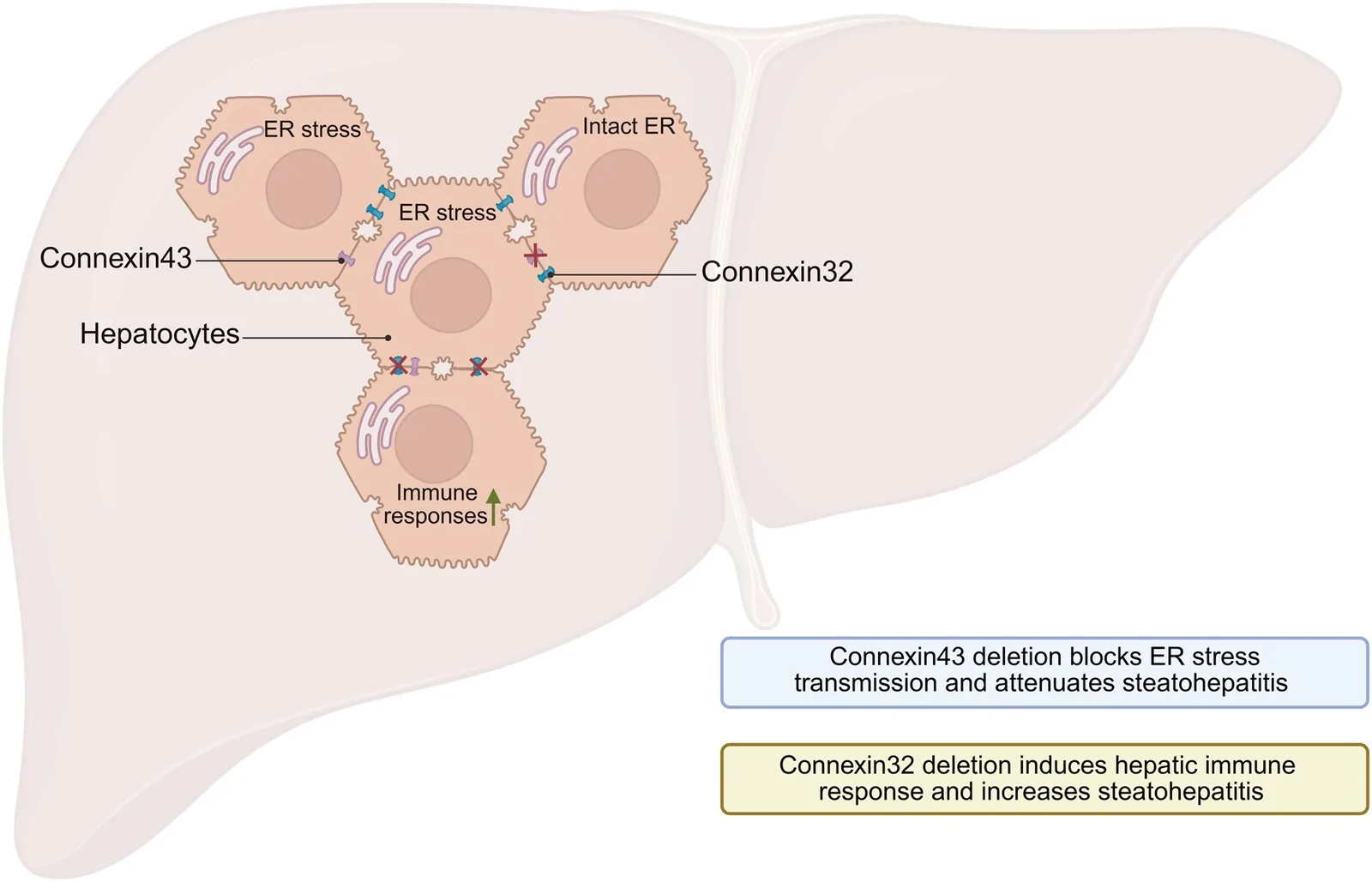
Connexins in the pathogenesis of metabolic MAFLD. Schematic illustration showing Cx43 and Cx32 exert different functions in the development of MAFLD.

As the major connexin expressed in hepatocytes, Cx32 shows a gradual decline during the progression of chronic liver diseases, including viral hepatitis, cirrhosis, and hepatocellular carcinoma in humans [104]. Emerging evidence suggests the role of hepatic Cx32 in the progression of MASLD, where Cx32 levels inversely correlate with the severity of MASLD in humans, including hepatic inflammation, ballooning, and fibrosis stages [105]. In Cx32-negative transgenic (Cx32ΔTg) rats, a marked impairment of gap junctional intercellular communication was found in the liver. When these mice were fed a methionine–choline-deficient diet, they exhibited exacerbated MASLD associated pathology, including more severe steatohepatitis and fibrosis, with increased hepatic levels of reactive oxygen species and inflammatory cytokines, compared to wild-type controls [106]. The number of GST-P-positive preneoplastic foci (preneoplastic lesions) was also significantly increased in Cx32ΔTg rats, indicating enhanced susceptibility to hepatocarcinogenesis [106]. Thus, Cx32 deficiency facilitates MASLD progression and the development of liver cancer [106]. Hepatic steatosis Cx32-deficient mice were similar to that of wild-type mice exposed to a choline-deficient HFD [107], suggesting that dietary methionine determines the effects of Cx32. However, Cx32^−^/^−^ mice exhibited more pronounced hepatic cell injury, as evidenced by elevated serum ALT, AST and IFN-γ levels, as well as increased hepatic expression of TNF-α and lipid peroxidation [107]. These findings suggest that Cx32 may influence hepatic immune responses, although the exact mechanisms remain to be elucidated (Fig. 7).

Connexin-mediated ER stress signals may also contribute to the development of MAFLD. Although ER stress is traditionally viewed as a cell-autonomous process, recent evidence shows that it can spread between hepatocytes, which depends on gap junction communications, because blocking Cx43 gap junctions with GAP27 or using Cx43-deficient hepatocytes can abolish the transmission of stress markers between hepatocytes, attenuate hepatic lipid accumulation, and improve glucose homeostasis (Fig. 7) [108

]. These findings suggest the potential of Cx43 as a therapeutic target to ameliorate MAFLD.

## Conclusions and perspectives

As our understanding of metabolic diseases deepens, the important roles of connexins in both health and disease have come into focus. Connexins, through their gap junctions and hemichannels, orchestrate a complex network of intercellular communication that is essential for maintaining tissue homeostasis and responding to metabolic stress. Moving forward, the therapeutic potential of targeting connexin-mediated signalling deserves closer attention. Modulation of specific connexin isoforms may offer a means to disrupt deleterious intercellular communication pathways while preserving or enhancing beneficial ones. However, given the diverse and context-dependent functions of connexins, a deeper mechanistic understanding is required to avoid unintended consequences. As research progresses, connexins may not only serve as biomarkers for the onset and progression of metabolic diseases but also as novel intervention points for halting or even reversing chronic metabolic complications. Thus, connexins stand at the crossroads of metabolic signalling, tissue health, and disease. Continued research into the molecular mechanisms and cell-specific actions of connexins will be essential for advancing our understanding of hypothalamic regulation and for developing new strategies to address metabolic disease.
